# Abiraterone, Orteronel, Enzalutamide and Docetaxel: Sequential or Combined Therapy?

**DOI:** 10.3389/fphar.2022.843110

**Published:** 2022-02-17

**Authors:** Ming-kun Chen, Zhi-jian Liang, Dao-Sheng Luo, Kang-yi Xue, De-ying Liao, Zheshen Li, Yuzhong Yu, Zhe-Sheng Chen, Shan-Chao Zhao

**Affiliations:** ^1^ Department of Urology, The Third Affiliated Hospital, Southern Medical University, Guangzhou, China; ^2^ Department of Urology, The Third Clinical College of Southern Medical University, Guangzhou, China; ^3^ Dongguan Hospital, Southern Medical University, Dongguan, China; ^4^ Department of Pharmaceutical Sciences, College of Pharmacy and Health Sciences, St. John’s University, Queens, NY, United States; ^5^ Department of Urology, Nanfang Hospital, Southern Medical University, Guangzhou, China

**Keywords:** metastatic castration-resistant prostate cancer, sequential therapy, combined therapy, docetaxel, abiraterone, orteronel, enzalutamide

## Abstract

**Objective:** To summarize the current therapeutic status using chemotherapeutic agent docetaxel and endocrine therapeutic agents (ARAT, abiraterone, orteronel or enzalutamide) for the treatment of metastatic castration-resistant prostate cancer (mCRPC), including sequential therapy and combined therapy, to promote the consensus on the optimal regimen for achieving superior treatment efficacy.

**Methods:** Through literature search in PubMed, articles with the following relevant keywords were collected and anlyzed: CRPC, abiraterone, orteronel and enzalutamide, median survival, overall survival, prostate specific antigen (PSA), PSA response rate and median radiologic progression-free survival.

**Results:** Fifty-eight articles were obtained and analyzed in this review. These articles included androgen axis-targeting agents after docetaxel, docetaxel after androgen axis-targeting agents, Triple sequential and combination therapy, covering four current drugs for mCRPC treatment: docetaxel, abiraterone, orteronel, and enzalutamide. It was found that there may be some cross-resistance between androgen axis-targeting agents, which will reduce the efficacy of subsequent drug treatment. Although neither of the studies of using combination therapy showed serious drug toxicity, the efficacy of sequential therapy was not as good as expected. Most adverse reactions after treatment were reported to be level 1–2.

**Conclusion:** Based on the results of the current studies, abiraterone followed by enzalutamide treatment is the best sequential treatment for most docetaxel-naïve patients. This treatment achieves not only good OS, but also PFS and PSA response rates. In addition, for patients who have previously failed docetaxel treatment, enzalutamide is the best choice as the subsequent treatment.

## Introduction

Prostate cancer is one of the most common malignancies among men in the world (Siegel et al., 2019). Since Huggins and Hodges (Huggins, 1941) discovered the effect of androgens on prostate cancer, androgen deprivation therapy (ADT) has became the main treatment for metastatic castration-sensitive prostate cancer (mCSPC) (Scher and Sawyers, 2005). However, although most patients can be relieved by ADT, most of them eventually progressed into castration-resistant prostate cancer (CRPC) (Bastos et al., 2014). The in-depth understanding of the mechanism of metastatic castration-resistant prostate cancer (mCRPC) led to the development of several new therapies, including taxanes (such as docetaxel) (Tannock et al., 2004), agents targeting androgen synthesis (such as abiraterone acetate and orteronel) (Potter et al., 1995; Yamaoka et al., 2012), and the androgen receptor inhibitor (enzalutamide) (Tran et al., 2009).

Docetaxel, one of the tubulin-binding taxanes, was the first chemotherapeutic agent demonstrated having survival benefits for mCRPC through two large randomized studies in 2004 (Table 1) (Petrylak et al., 2004; Tannock et al., 2004). Subsequently, the androgen receptor (AR) was gradually understood. AR is a nuclear hormone receptor, which depends on the activation of dihydrotestosterone and a ligand produced by the intracellular conversion of testosterone, which induces nuclear localization and target gene transcription (Lu et al., 2006). The emergence of new endocrine agents including abiraterone, orteronel and enzalutamide have also been added to the treatment options for mCRPC. Among them, abiraterone is a potent and irreversible cytochrome inhibitor that inhibits androgen synthesis and acts as an androgen receptor antagonist (Richards et al., 2012). Similarly, Orteronel inhibits androgen synthesis by selecting CYP17, 20-lyase as a reversible inhibitor (Kaku et al., 2011; Yamaoka et al., 2012). Followed by enzalutamide, which is a second-generation antiandrogen agent developed on the basis of a preclinical model of bicalutamide resistance with an androgen receptor mutation or overexpresses the androgen receptor (Hoffman-Censits and Kelly, 2013). Both endocrine therapies have been shown to improve overall survival of mCRPC either as a single medicine or combined with others.

**TABLE 1:** Phase 3 Trials in Metastatic Castration-Resistant Prostate Cancer.

Trials	Trial Registration No. (Clinical Trials. gov Identifier)	Sample Size			Treatment		Median OS (Months)			Median time to PSA progression (Months)			PSA response (%)			median radiologic progression-free survival (Months)		
		Control	Size treatment	Total	Control	Size treatment	HR	Control	Study treatment	HR	Control	Study treatment	HR	Control	Study treatment	HR	Control	Study treatment
TAX 327 (2004)(Tannock et al., 2004)		D30: 334 D30: 334	335	1006	Mitoxantrone 12 mg/m2 every 3 weeks (M) Docetaxel 30 mg/m2 per week (D30)	Docetaxel 75 mg/m2 every 3 weeks (75)	D75 : M: 0.74 (95% CI NR)	M: 16.5 (95% CI 14.4-18.6) D30: 17.4 (95% CI 15.7-19.0)	18.9 (95% CI 17.0-21.2)	NR	NR	NR	NR	M: 50% PSA decrease: 96(32%) D30: 50% PSA decrease: 135(48%)	50% PSA decrease: 131(45%)	NR	NR	NR
SWOG 99–16 (2004)(Petrylak et al., 2004)		336	338	674	Mitoxantrone 12 mg/m2 every 3 weeks	Docetaxel 60 mg/m2 (day 1) plus estramustine 260 mg (days 1–5) every 3 weeks	0.80 (95% CI 0.67-0.97; p=0.02)	15.6 (95% CI NR)	17.5 (95% CI NR)	P<0.001	3.2 (95% CI NR)	6.3 (95% CI NR)	P<0.001	50% PSA decrease: 83(27%)	50% PSA decrease: 155(50%)	NR	NR	NR
COU-AA-301 (2012)(Fizazi et al., 2012)	NCT00638690	398	797	1195	Placebo plus prednisone (5 mg, twice daily)	Abiraterone acetate (1000 mg once a day) plus prednisone (5 mg twice a day)	0.74 (95% CI 0.64–0.86; p<0.0001)	11.2 (95% CI 10.4–13.1)	15.8 (95% CI 14.8–17.0)	0.63 (95% CI 0.52−0.78; p<0.0001)	6.6 (95% CI 5.6–8.3)	8.5 (95% CI 8.3–11.1)	NR	50% PSA decrease: 22 (5.5%)	50% PSA decrease: 235 (29.5%)	0.66 (95% CI 0.58−0.76; p<0.0001)	3.6 (95% CI 2.9–5.5)	5.6 (95% CI 5.6–6.5)
COU-AA-302 (2013)(Ryan et al., 2013)	NCT00887198	542	546	1088	Placebo plus prednisone (5 mg twice a day)	Abiraterone acetate (1000 mg once a day) plus prednisone (5 mg twice a day)	0.75 (95% CI 0.61-0.93; P=0.01)	27.2 (95% CI NR)	NR	0.49 (95% CI 0.42–0.57; p<0.0001)	5.6 (95% CI NR)	11.1 (95% CI NR)	2.59 (95% CI 2.19–3.05; p<0.0001)	50% PSA decrease: 130(24%)	50% PSA decrease: 338(62%)	0.53 (95% CI 0.45-0.62; P<0.001)	8.3 (95% CI NR)	16.5 (95% CI NR)
AFFIRM (2012)(Scher et al., 2012)	NCT00974311	399	800	1199	Placebo	Enzalutamide (160 mg once a day)	0.63 (95% CI 0.53-0.75; P<0.001)	13.6 (95% CI 11.3-15.8)	18.4 (95% CI 17.3-NR)	0.25 (95% CI 0.20-0.30; p<0.001)	3.0 (95% CI 2.9-3.7)	8.3 (95% CI 5.8-8.3)	NR	50% PSA decrease: 5(2%)	50% PSA decrease: 395(54%)	0.40 (95% CI 0.35-0.47; P<0.001)2.9 (95% CI 2.8-3.4)	2.9 (95% CI 2.8-3.4)	8.3 (95% CI 8.2-9.4)
PREVAIL (2014)(Beer et al., 2014)	NCT01212991	845	872	1717	Placebo	Enzalutamide (160 mg once a day)	0.73 (95% CI 0.63-0.85; P<0.001)	31.0 (95% CI NR)	NR	0.17 (95% CI 0.15–0.20; P<0.001)	2.8 (95% CI NR)	11.2 (95% CI NR)	NR	50% PSA decrease: 27(3%)	50% PSA decrease: 666(78%)	0.19 (95% CI 0.15-0.23; P<0.001)	3.9 (95% CI NR)	NR
ELM-PC 4 (2015)(Saad et al., 2015)	NCT01193244	779	781	1560	Placebo (400 mg) plus prednisone (5 mg twice daily)	Orteronel (400 mg) plus prednisone (5 mg twice daily)	0.80 (95% CI 0.64-0.99; p=0.043)	29.5 (95% CI 27.0-NR)	31.4 (95% CI 28.6-NR)	NR	NR	NR	<0·0001	50% PSA decrease: 192(25%)	50% PSA decrease: 333(43%)	0.71 (95% CI 0.63-0.80; p<0.001)	8.7 (95% CI 8.3-10.9)	13.8 (95% CI 13.1-14.9)
ELM-PC 5 (2015)(Fizazi et al., 2015)	NCT01193257	365	734	1099	Placebo (400 mg) plus prednisone (5 mg twice daily)	Orteronel (400 mg) plus prednisone (5 mg twice daily)	0.886 (95% CI 0.739- 1.062; P=0.190)	15.2 (95% CI 13.5-16.9)	17 (95% CI 15.2-19.9)	0.698 (95% CI 0.602-0.809; P<0.001)	2.9 (95% CI 2.83-2.89)	5.5 (95% CI 4.4-5.56)	NR	NR	NR	0.76 (95% CI 0.653-0.885; P<0.001)	5.7 (95% CI 5.5-7.0)	8.3 (95% CI 7.8-8.5)

Abbreviations: 95% CI, 95% confidence interval; OS, overall survival; PSA, prostate-specific antigen; NR, not reported.

However, there were controvercial about the sequential or combined treatment strategy. The presence of a two-compartment, potentially adaptive, feedback loop in single-agent studies of androgen blockers suggests that combination therapies may eliminate adaptive responses between the respective drugs (Efstathiou et al., 2012; Efstathiou et al., 2015). Compared to traditional sequential therapies, a new drug is changed until the prodrug fails, they believe that simultaneous combination use may help improve patient outcomes (Attard et al., 2018; Efstathiou et al., 2020). Thus, we summarize the current and ongoing clinical studies on the sequences and combination of these agents for the treatment of mCRPC in this review.

## Methods

Relevant keywords involving mCRPC, docetaxel, abiraterone, orteronel, and enzalutamide were used for literuature search in Pubmed. The median survival was recognized as the primary outcome, and the median time to PSA progression, PSA response and median radiologic progression-free survival were defined as secondary outcomes. After constructing the literature pool and extracting the above results of patients with various treatment strategies that were available, the results were compared with the corresponding large randomized Phase III study (Table 1).

## Results

**FIGURE 1:**
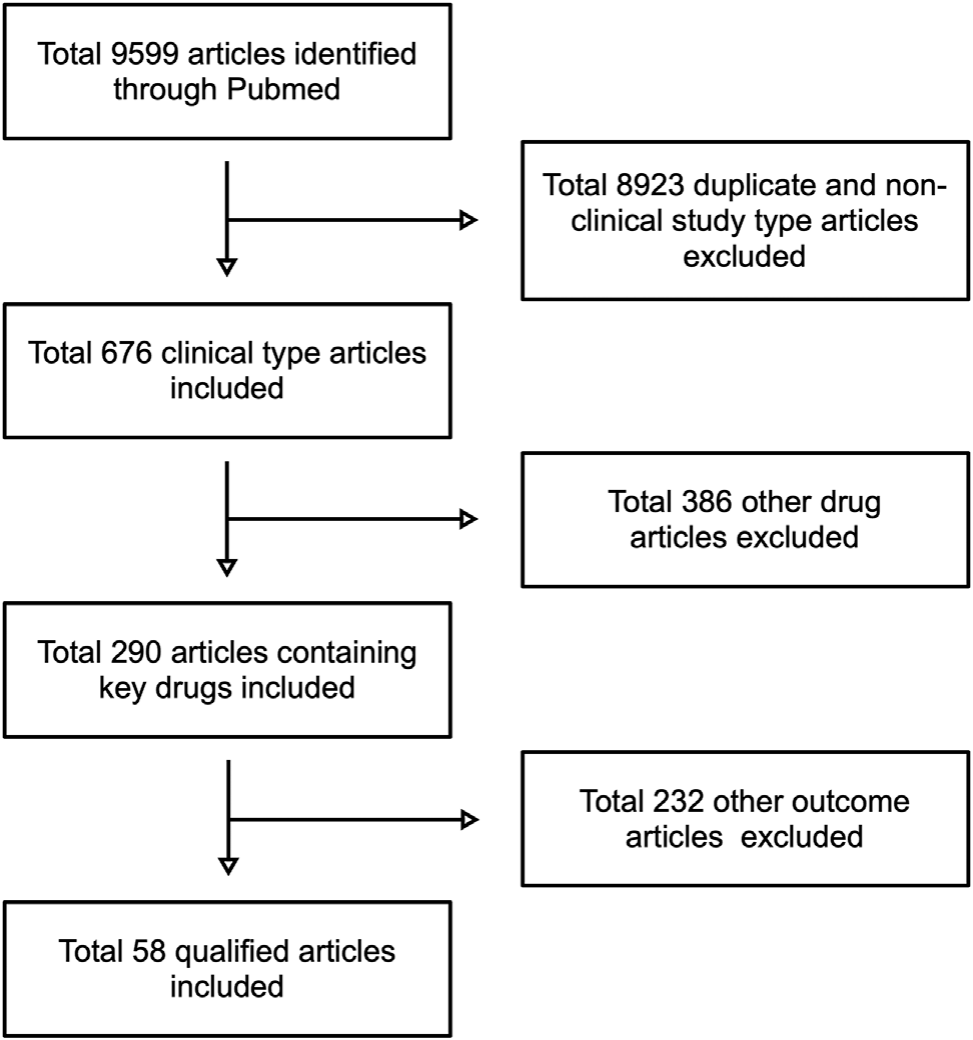
The screen and filtering processes for this study.

The flow-chart for the retrieval process is shown in Figure 1. A total of 5,481 publications were identified by PubMed by search keywords, which were then filtered according to the content of the articles, resulting in a total of 58 compliant articles.

### Chemotherapy After Androgen Axis-Targeting Agents

**FIGURE 2:**
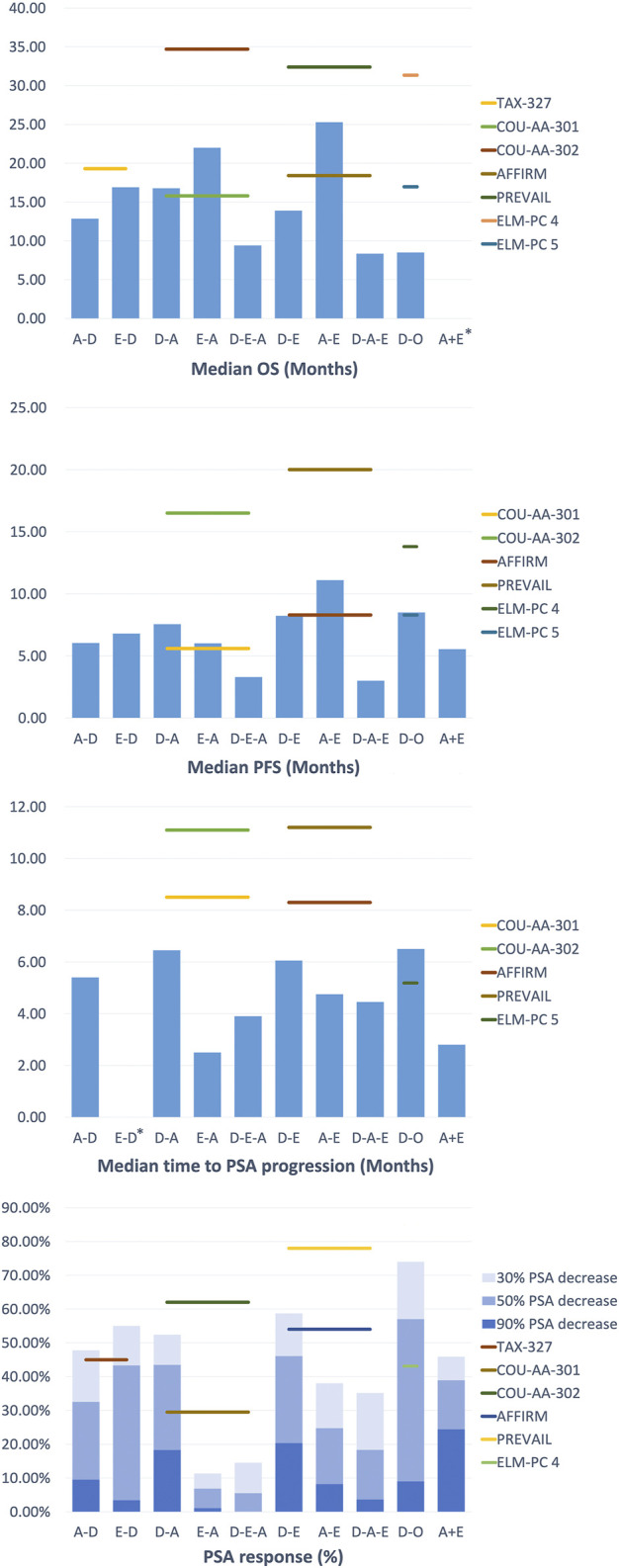
Clinical Outcomes of treatment strategy in Metastatic Castration-Resistant Prostate Cancer.

For those who had previously treated with abiraterone followed by docetaxel (A-D), Schweizer et al. described up to 100% of patients with Eastern Cooperative Oncology Group (ECOG) performance status (PS) 0–1 (Schweizer et al., 2014). By comparison, the PSA decline of ≥50% (38% vs 63%; p = 0.02), median progression-free survival (PFS) (4.4 months vs 7.6 months; p = 0.003), and median prostate-specific antigen progression-free survival (PSA-PFS) (4.1 month vs 6.7 months; p = 0.002) in the A-D group were lower than those in the D group (only experienced docetaxel) (Supplementary Table S1, Supplementary Figure S1). This indicated that the progression risk for the A-D group may be higher than that of Group D and is unlikely to receive a PSA response (Figure 2). In contrast, the median OS and median PFS of patients in the studies with a relatively low proportion of ECOG PS 0–1 were not significantly affected, suggesting that cross-resistance may be absent between the two agents (Azad et al., 2014; Miyake et al., 2017). Combined with different levels of PSA baselines in different retrospective studies, A-D sequential therapy may be more suitable for patients with advanced disease and large tumor burden.

For patients who were treated with enzalutamide followed by docetaxel (E-D), Miyake et al. reported similar results to the TAX-327 experimental group, suggesting that there may be no cross-resistance in enzalutamide and docetaxel. At the same time, they performed multivariate analyses to determine the independent prognostic indicator: PS for PSA PFS and PS and visceral metastasis for OS. The findings indicated that as mCRPC patients progress with enzalutamide, the introduction of docetaxel was best in patients with favorable performance status (PS) to maximize prognostic benefits. Finally, docetaxel was well tolerated, and no unintended toxic side effects are reported in A-D, while specific side effects of this sequential therapy have not been reported in E-D.

### Androgen Axis-Targeting Agents After Docetaxel

Treatment with docetaxel before abiraterone (D-A) generally shows satisfactory effect and an acceptable safety profile, but the subsequent improvement in treatment results is lacking.

As in a multi-center study by Satoh et al., the 50% PSA reduction rate for mCRPC patients at 3 months was 28.3%, and the median OS at 6 months was 89.1%. Additionally, the ≥50% PSA decline rate was 48.4%, the median OS and PFS were 30.2 and 7.3 months in the study of Chang et al. (Chang et al., 2019), which is similar to the outcomes (≥50% PSA decrease in 51.6%, median OS in 24 months and PFS in 6.6 months) of the study of Li et al. (Li et al., 2017). These results were better than that in the D-A sequential treatment in COU-AA-301 (29.5%, 15.8 months, 5.6 months, respectively), yet they were inferior to COU-AA-302 (62%, not reported, 16.5 months, respectively). This may be due to the basic characteristics of the patients in the above study (100% ECOG PS 0–1) and the longer follow-up time, which leads to a more extended OS shift. Furthermore, in the study of Lin et al. (Lin et al., 2019), D-A sequence also appeared to be inferior compared to those who have not previously received D treatment (median OS in 18 vs 27 months, *p* = 0.016, median rPFS in 12.5 vs 17 months, *p* = 0.003), revealing the possible cross-resistance between docetaxel and abiraterone.

In the treatment of docetaxel followed by enzalutamide (D-E), Noonan et al. and Chang et al. found that the ≥30% PSA reduction rates were as high as 70 and 76.9%, the ≥50% PSA reduction rates were 60 and 69.2%, and the median PFS was 11.9 and 9.5 months respectively (Noonan et al., 2013; Chang et al., 2019). There was no significant difference between the results of the two studies. However, compared with the other group of D-A sequential therapy in their respective studies, the D-E therapy seemed to be slightly better in maintaining PSA response rate and PSA decline, except for PFS and OS (Cheng et al., 2015). Furthermore, in the docetaxel followed by orteronel (D-O), Cathomas et al. concluded that orteronel significantly lengthens EFS in patients achieving disease stabilization (median EFS in 8.5 vs 2.9 months, *p* = 0.001), but other aspects are slightly inferior to other sequential treatments (Cathomas et al., 2016).

The above results indicated that there was no significant difference in efficacy in sequential therapy. Still, D-A sequential therapy can achieve better results in PSA response rate and PFS for patients with mCRPC. The most common adverse events were fatigue, pain, and anemia.

### Sequential Treatment With Androgen Axis-Targeting Agents

In the study of the therapeutic effect relationship between androgen axis-targeting agents, two sequential treatment groups were established: abiraterone followed by enzalutamide treatment (A-E) and the opposite order (E-A). This study design provided a better understanding of two AR-axis targeted (ARAT) drugs, with the opposite order for therapeutic effects and the possibility of cross-resistance. Interestingly, in the current research reports, the conclusions of each study are strikingly consistent.

Studies by Terada et al. and Komura et al. showed that enzalutamide remained effective when used as a second-line drug following abiraterone treatment (Terada et al., 2017; Komura et al., 2019). However, in the sequential treatment of E-A, the PSA response of abiraterone as a second-line treatment was significantly lower than the experimental group in COU-AA-301 (≥50% PSA decline rate in 29.5%) and COU-AA-302 (≥50% PSA decline rate in 62%). On the other hand, the PSA response of A-E sequence with better healing effect also decreased to a certain extent compared with AFFIRM (≥50% PSA decline rate in 54%) and PREVAL (≥50% PSA decline rate in 78%) (Azad et al., 2015; Attard et al., 2018; Khalaf et al., 2019). These results revealed the possibility of cross-resistance between abiraterone and enzalutamide.

Overall, these studies revealed the cross-resistance between abiraterone and enzalutamide, and their efficacy was similar in the first-line treatments. Still, the A-E sequence was more effective in second-line PFS and combined PFS, even if there was no difference observed in OS. The most common level 3–4 adverse event in the treatment is hypertension, but no treatment-related deaths have occurred.

### Triple Sequence

In the third-line sequential therapy, only a few studies evaluated the efficacy of androgen blockers as third-line therapy in mCRPC patients. After undergoing docetaxel and enzalutamide sequential therapy plus abiraterone (D-E-A), Noonan et al. found that although 70% of patients had a ≥30% PSA decrease in enzalutamide, only 11 and 3% of patients had at least a ≥30% and ≥50% PSA decrease in subsequent treatment with abiraterone acetate (Noonan et al., 2013). The median PFS and duration of abiraterone acetate treatment were short, at 3.9 months (95% CI 2.7–5.1) and 3 months (95% CI 0.3–12.8), respectively. Similarly, the results from the study of Loriot et al. were generally not satisfactory (Loriot et al., 2013). In contrast, when enzalutamide was lastly used (D-A-E), the results of studies such as Thomsen et al. and Badrising et al. were broadly consistent (Badrising et al., 2014; Bianchini et al., 2014; Thomsen et al., 2014). They showed that the outcomes were slightly less than in the D-E group in AFFIRM (50% PSA decrease: 54%, the median PFS: 8.3 months), revealing the heterogeneity of prostate cancer in terms of AR signaling addiction and drug resistance.

Interestingly, the above study suggested that some patients who were sensitive to the primary AR axis inhibitors may maintained a degree of response to subsequent treatment. Therefore, although there may be cross-resistance between abiraterone and enzalutamide as a whole, for some patients who progress after enzalutamide treatment, certain sensitivity to abiraterone may remain. Further prospective studies of prostate cancer heterogeneity are particularly important to assess the sequencial use of abiraterone and enzalutamide to help determine the best sequencing of using these drugs.

Additionally, in a prospective study by Schmid et al., only 10% of patients achieved a ≥50% reduction in PSA response, with a median OS of 7.5 months and a median PFS of 3.1 months (Schmid et al., 2014). This prospective study suggested that in the sequential treatment of D-A-E, the clinical effect of enzalutamide was more modest than its role in early tumor stage. They concluded that the preclinical results did not support the continuous use of abiraterone and enzalutamide. They proposed that a reasonable treatment strategy may be the alternating use of chemotherapy and antihormonal drugs, such as A-D-E.

Furthermore, to verify the impact of the treatment of previous docetaxel on subsequent treatment, Azad et al. set up two groups of treatment using D-A-E and A-E (Azad et al., 2015). They found that the efficacy of enzalutamide was comparable between the two groups, with similar PSA response rates (22 vs 26%, *p* = 0.8), median radiology/clinical time to progression (4.6 vs 6.6 months, *p* = 0.6) and median OS (10.6 vs 8.6 months, *p* = 0.2). The efficacy of enzalutamide was similar regardless of previous docetaxel use, suggesting that the cross-resistance between abiraterone and enzalutamide was independent to docetaxel. Although the activity of enzalutamide was limited, 21% of patients in the study remained enzalutamide-sensitive for at least 6 months, demonstrating that the D-A-E sequential treatment regimen could provide long-term benefits for patients. In general, there is a certain degree of cross-resistance between abiraterone and enzalutamide. Regardless of whether or not docetaxel has been used, the overall activity of abiraterone and subsequent enzalutamide treatment is limited, but some patients can still get long-term benefits. The need to develop reliable predictive biomarkers to identify these patients is critical. Patients treated with triple sequences were well tolerated, had no accidental toxicity, and most adverse reactions were grades 1–2.

### Combination Therapy

The studies of combined treatment that can be retrieved in the present study is between androgen blockers (A + E), and the outcomes of the search was quite different. For example, in the study by Attard et al., the median PFS was only 7.5 months, the ≥30% and ≥50% PSA decline rates were 4.8 and 0.8%, respectively (Attard et al., 2018) (Supplementary Table S2). In contrast, Efstathiou et al. reported the 50% PSA decline rate was as high as 77% and the median PFS was 8.4 months, better than the results of Attard et al. (Efstathiou et al., 2020). However, this might be related to the characteristics and previous medications of the patients. The proportion of patients with a Gleason score of ≥8 in the study of Attard et al. was as high as 92.5%, and the median age was 72 years, which was higher than that of the survey by Efstathiou et al. Moreover, the patients in the study by Attard et al. had previously received the treatment of enzalutamide and were treated with combination therapy until the disease progressed.

In addition, COU-AA-302 and PREVAIL seem to take longer than the time reported above in terms of time to progress and 50% PSA decline (11.1 months, 24%; 11.2 months, 78%). Both studies suggested that the results of the efficacy study do not support the treatment plan of abiraterone combined with enzalutamide. Although the combination therapy was not as good as expected, some patients in the study by Efstathiou et al. achieved excellent results. This may be because inhibition of AR and androgen biosynthesis may further prolong the survival of some mCRPC patients by delaying the emergence of drug resistance. Therefore, finding and verifying biomolecular markers is necessary to identify patients who will benefit from the combination therapy.

No severe drug toxicity occurred in either study, reflecting the safety of the combined use of the two drugs.

## Discussion

Over the past 10 years, tremendous changes have taken place in the treatment strategy of mCRPC, especially after the emergence of many new drugs and their clinical application. The traditional docetaxel chemotherapy has obvious limitations, such as requiring patients with high PS, moderate survival benefits, and relatively serious side effects. But docetaxel has its own unique advantages: the course of treatment is shorter than that of abiraterone and enzalutamide, the total cost is low, and glucocorticoids are not required (Caffo et al., 2011; Al-Batran et al., 2015). On the other hand, the new ARAT agents, abiraterone, orteronel and enzalutamide, have been proven to have considerable survival benefits, with low requirements for the patient’s PS condition. At the same time, the side effects caused by the agents are not obvious compared to docetaxel (de Bono et al., 2011; Scher et al., 2012; Ryan et al., 2013; Beer et al., 2014). Multiple studies have shown that subsequent ARAT agents are somewhat less effective during sequential D-ARAT treatment, suggesting that previous docetaxel chemotherapy may produce resistance to AR (Esch et al., 2014; Lorente et al., 2015). Since there was no study on the median OS reported in the A + E combination therapy, it is not possible to compare with sequential treatments at the median OS level. However, Attard et al. found that there was no significant difference in PFS by comparing A + E and E-A (5.7 vs 5.6 months, *p* = 0.22), and the frequency of grade 3 hypertension (10 vs 2%) and increased ALT (6 vs 2%) or AST (2 vs 0%) was more frequent in the A + E group. Current studies on combination therapies have been unable to support substantial efficacy benefits. For the time being, different combination therapy trials for mCRPC are ongoing (Table 2), which is expected to continue to supplement the vacuum in this field. Therefore, ARAT agents are recommended as a first-line treatment for mCRPC, rather than docetaxel or combination therapy.

**TABLE 2:** Ongoing Clinical Trials in Metastatic Castration-Resistant Prostate Cancer

Registration No. (ClinicalTrials.gov Identifier)	Start Date	Phase	Sample size	Interventions		Endpoints	Description
				Arm A	Arm B		
NCT02036060	January 2014	2	119	Docetaxel plus abiraterone	Docetaxel	OS, PSA response, rPFS, quality of life,	Abiraterone in combination with docetaxel after disease progression to abiraterone
NCT02125357	April 2014	2	202	First line: abiraterone Second line: Enzalutamide	first line: enzalutamide second line: abiraterone	PSA response	Sequencing of abiraterone and enzalutamide (or vice versa)
NCT03419234	February 2018	2	210	Abiraterone plus cabazitaxel	abiraterone	OS, PFS, TTPP	Abiraterone and ADT with or without cabazitaxel and prednisone in patients previously treated with docetaxel
NCT03896984	April 2019	2	300	First line: abiraterone or enzalutamide Second line: Radium-223	First line: Abiraterone Second line: Enzalutamide (or vice versa)	OS	Novel anti-hormone therapy followed by a second line treatment with novel anti-Hormone therapy or radIum-223
NCT02288247	November 2014	3	690	Docetaxel plus enzalutamide	Docetaxel plus placebo	ORR, PFS, PSA response, TTPP	Enzalutamide in combination with docetaxel after disease progression to enzalutamide
NCT01949337	September 2013	3	1311	Enzalutamide	Enzalutamide plus abiraterone	OS, ORR, PFS, PSA response, Grade 3 or higher toxicity profile	Enzalutamide with or without abiraterone and prednisone
NCT03641560	August 2018	4	52	Enzalutamide	—	PSA response, Safety assessed by incidence of adverse events,	Enzalutamide in patients previously treated with docetaxel
NCT02485691	June 2015	4	324	Cabazitaxel	Abiraterone or enzalutamide	OS, rPFS PSA response	Cabazitaxel versus the switch to alternative ar-targeted agent (enzalutamide or abiraterone) in patients previously treated with docetaxel
NCT01995513	November 2013	4	509	Abiraterone plus enzalutamide	Abiraterone plus placebo	ORR,PFS, PSA response	Continued enzalutamide with abiraterone beyond progression on abiraterone

The effect of the sequence of A and E is relatively better than that of the ARAT-D sequence, especially in OS. Also, when ARAT agents were used as the first-line treatment, komura et al. showed that there was no significant difference in efficacy between abiraterone and enzalutamide (Komura et al., 2019). However, in the second-line treatment, A-E is superior to E-A in PFS (15 vs 7 months, *p* = 0.04), time to PSA progression (TTPP; 6 vs 3 months, *p* = 0.008) and PSA response (*p* = 0.01), except that OS did not reach significance (14 vs 23 months, *p* = 0.35).

A meta-analysis to compare oncologic outcomes between the treatment sequences of A-E and E-A by Chung et al. also came to similar conclusions (Chung et al., 2019). They reported that the former could obtain better outcomes in PFS than the latter (*p* < 0.0001), especially in docetaxel-naïve CRPC patients, except for OS (*p* = 0.10). The adverse event rate of grade 3 or worse was roughly similar between A-E and E-A. Therefore, according to the current research results, A-E sequential therapy is the best choice for patients with mCRPC.

The selection of the best treatment strategy for mCRPC patients, in addition to focusing on the best efficacy, other factors should also be considered, such as economic conditions and comorbidities et al. Fortunately, the total cost of A-E is lower in sequential treatment of ARAT on the premise of maintaining relatively superior efficacy. Because the use of drug treatment as the first-line treatment may have the longest duration until the disease progresses, and it costs the most, abiraterone might be more cost-effective than enzalutamide and docetaxel. Abiraterone is not applicable if the patient has a contraindication to the use of glucocorticoids.

For patients who have previously failed docetaxel, whether ARAT drugs could be used as the next line of treatment is another critical issue. In the D-ARAT sequential therapy, Chang et al. reported that D-E group is slightly better than D-A group on PSA response rate (≥50% PSA decrease: 69.2 vs 48.4%, *p* = 0.171; ≥90% PSA decrease: 38.5 vs 25%, *p* = 0.320) and median PFS (9.5 vs 7.3 months, *p* = 0.734). In Fang et al., a trial-level meta-analysis, also showed no significant statistical difference in OS, although D-E was 2.2 months more than D-A (Fang et al., 2017). In another assessment analysis of the efficacy in the study of Chopra et al., the efficacy of abiraterone and enzalutamide was indirectly compared in both the pre-docetaxel and post-docetaxel settings, based on published phase III randomized trials (Chopra et al., 2017). It is concluded that enzalutamide outperforms abiraterone in terms of PSA response rate, median PFS and TTPP. D-O is currently known to have fewer studies, only reported significantly prolonged EFS in patients with stable disease, the rest of the results were not significantly prominent. The adverse event rate of grade 3 or worse was roughly similar in androgen axis-targeting agents. Therefore, enzalutamide as the next treatment agent i.e. D-E, may be a more reasonable and effective choice in patients who fail after treatment with docetaxel.

Additionally, in a prospective study by Schmid et al., only 10% of patients achieved a ≥50% reduction in PSA response, with a median OS of 7.5 months and a median PFS of 3.1 months. This prospective study demonstrated that the clinical effect of enzalutamide is modest using the sequential treatment of D-A-E. They concluded that the preclinical results do not support the continuous use of abiraterone and enzalutamide. They proposed that a reasonable treatment strategy may be the alternating use of chemotherapy and antihormonal drugs, such as A-D-E. Although there is no other prospective study to confirm this result, and this trial has limitations such as small sample size and no random design, an appropriate sequencial treatment may include A-D-E. In addition to AR signaling conduction in mCRPC, poly (ADP-ribose) polymerase inhibitors (PARPi) for the treatment of defective tumors is of great interest. Among them, the new drugs niraparib and olaparib both showed anti-tumor activity against metastatic deaggressive prostate cancer with abnormal DNA damage response (DDR) gene(Smith et al., 2019; Mateo et al., 2020). In the future, PARPi may become the first choice of targeted therapy for mCRPC.

Most of the current studies are limited to retrospective features, and there might be some biases compared to the real world. To date, there are no high-level research data to prove and support any scheme related to mCRPC treatment. Therefore, to select the most suitable and effective treatment strategy based on the essential characteristics of each patient, further high-quality clinical research is urgently needed.

## Conclusion

The research in the treatment of mCRPC in the past 10 years has played an essential role in promoting the choice of the most effective treatment method according to individual patients. Based on the results of the current studies, A-E may be the best sequential treatment for most docetaxel-naïve patients. This treatment has good feedback not only on OS, but also on PFS and PSA response rates. In addition, for patients who have previously failed docetaxel treatment, enzalutamide is the best choice for the next stage of treatment.
