# Supplementary material for: Abiraterone, Orteronel, Enzalutamide and Docetaxel: Sequential or Combined Therapy?
Source: Front Pharmacol. 2022 Feb 17;13:843110. doi: 10.3389/fphar.2022.843110 (PMC8891580; doi:10.3389/fphar.2022.843110)
Supplement: Supplementary file 1 [file Table1.docx]

Supplemental table 1. Clinical Outcomes of Sequential Treatment in Metastatic Castration-Resistant Prostate Cancer

|  | **Sample Size** | **Median OS (Months)** | **Median time to PSA progression (Months)** | **PSA response (%)** | | | **Median PFS (Months)** |
| --- | --- | --- | --- | --- | --- | --- | --- |
|  |  |  |  | **30% PSA decrease** | **50% PSA decrease** | **90% PSA decrease** |  |
| **Abiraterone after docetaxel (D-A)** | | | | | | | |
| Thomsen et al. (2013)^34^ | 24 | 4.8 (95% CI 3.0-8.4) | NR | 14(58.3%) | 8(33.3%) | 1(4.2%) | NR |
| Bianchini et al. (2014)^37^ | 39 | NR | NR | 19(48.7%) | 15(38.4%) | 6(15.3%) | NR |
| Schrader et al. (2014)^46^ | 35 | NR | NR | 17(48.6%) | 16(45.7%) | 10(28.6%) | NR |
| Satoh et al. | 47 | 6-month OS:  89.1% (95% CI 78.6-94.6%) | NR | 15(32.6%) | 13(28.3%) | 2(4.3%) | NR |
| Sonpavde et al. (2015)^47^ | 183 | 17 (95% CI 14-NR) | NR | NR | NR | NR | NR |
| Maughan et al. (2015)^48^ | 26 | 19.4 (95% CI NR) | NR | NR | NR | NR | 9.5 (95% CI NR) |
| Sun et al. | 143 | 12-month OS:  77.1% (68.1%-83.9%) | 5.6(95% CI 4.7-6.6) | NR | NR | NR | NR |
| Cicero et al. | 60 | 14 (95% CI 12.9-15.0) | NR | NR | NR | NR | 5 (95% CI 4.4-5.6) |
| Li et al. | 64 | 24 | NR | NR | 33(51.6%) | NR | 6.6 |
| Chang et al. | 63 | 30.2 (95% CI NR) | 7.3 (95% CI NR) | 34(54.0%) | 31(48.4%) | 16 (25%) | 7.3 (95% CI 4.8-9.8) |
| Puente et al. | 100 | NR | NR | NR | NR | NR | 9.2 |
| Lin et al. | 43 | 8 | NR | NR | NR | NR | rPFS: 12.51  PSA-PFS: 7.7 PSA-PFS: 7.7 |
| **Enzalutamide after Docetaxel (D-E)** | | | | | | | |
| Noonan et al. | 30 | NR | NR | 21(70%) | 18(60%) | 7(23%) | 11.9 (95% CI 7.1-15.8) |
| Nadal et al. | 60 | 11.6 (95% CI 8.7-15.2) | 2.6 (95% CI 1.9-3.5) | 27(45%) | 15(25%) | 4(6.7%) | 3.3 (95% CI 2.5-4.8) |
| Cheng et al. | 30 | 12-month OS:  77% (61%–97%) | NR | 13(43%) | 9(30%) | 4(13%) | PSA-PFS: 4.1 (95% CI 2.9–5.4) |
| Chang et al. | 13 | 16.2 (95% CI NR) | 9.5 (95% CI NR) | 10(76.9%) | 9 (69.2%) | 5 (38.5%) | 9.5 (95% CI 5.7-13.3) |
| **Enzalutamide after Orteronel (D-O)** | | | | | | | |
| Cathomas et al. | 23 | 6.5 (95% CI 2.7-10.3) | NR | 17(74%) | 13(57%) | 2(9%) | rPFS: 8.5 (95% CI 3.5-14.2) |
| **Docetaxel after abiraterone (A-D)** | | | | | | | |
| Mezynski et al. (2012)^16^ | 35 | 12.5 (95% CI 10.6–19.4) | 4.6 (95% CI 4.2-5.9) | 13 (37%) | 9(26%) | 0(0%) | NR |
| Schweizer et al. (2014)^17^ | 24 | NR | NR | 13 (54%) | 9 (38%) | 1(4.2%) | 4.4 (95% CI 3.1–6.7)  PSA-PFS: 4.1 (95% CI 2.8-5.8) |
| Aggarwal et al. (2014)^53^ | 23 | 12.4 (95% CI 8.2–19.6) | NR | 15(65%) | 11(48%) | 6(26.1%) | NR |
| Azad et al. | 86 | 11.7 (95% CI 9.5–13.9) | 4.0 (95% CI NR) | 41(47.7%) | 30 (35%) | 5(5.8%) | 4.0 (95% CI 3.1–5.0) |
| Ueda et al. | 15 | 14.4 （95% CI 6.3-22.4） | NR | 5(33%) | 2(13%) | 0(0%) | 3.7 (95% CI 2.9-4.6) |
| Maughan et al. (2015)^48^ | 32 | 7.3 | NR | NR | NR | NR | 10.4 (95% CI NR) |
| Miyake et al. | 54 | 18.8 (95% CI NR) | NR | 27(50%) | 22(40.7%) | 1(1.9%) | 7.7 (95% CI NR) |
| Bono et al. | 261 | NR | 7.6 (95% CI 5.0-NR) | NR | 27(27%) | NR | NR |
| **Enzalutamide after abiraterone (A-E)** | | | | | | | |
| Cheng et al. | 79 | 12-month OS:  64% (45%–90%) | NR | 22(28%) | 14(18%) | 2(3%) | PSA-PFS: 4.0 (95% CI 3.2–4.8) |
| Azad et al. | 115 | 10.6 (95% CI NR) | NR | 40(35%) | 27 (24%) | 5(4%) | NR |
| Maughan et al. (2016)^56^ | 65 | 33.3 (95% CI 25.4-NR) | NR | 31(47.7%) | 25(38.5%) | 9(13.8%) | 19.5 (95% CI 15.5-22.3) PSA-PFS: 17.5 (95% CI 14.0-19.5) |
| Terada et al. | 113 | 30.6 (95% CI 25.4-NR) | NR | 44(43.1%) | 30(29.4%) | 12(11.8%) | PSA-PFS: 15.2 (95% CI 12.8-16.5) |
| Matsubara et al. (2017)^57^ | 50 | 25.4 (95% CI 19.8-31.1) | NR | NR | 9(18%) | NR | 11.1 (95% CI 8.1-14.1) |
| Emamekhoo et al. (2018)^58^ | 40 | NR | NR | 12(30%) | 8(20%) | 3(7.5%) | PSA-PFS: 2.3 (95% CI 1.8-3.4) |
| Khalaf et al. | 73 | 28.8 (95% CI 25.4-NR) | 3.5 (95% CI 2.9-8.7) | 26(35.6%) | 17(23.3%) | 6(8.2%) | NR |
| Komura et al. | 46 | 23 (95% CI NR) | 6 (95% CI NR) | 21(46.7%) | 12(26.7%) | 4(8.9%) | rPFS: 15 (95% CI NR) |
| **Docetaxel after enzalutamide (E-D)** | | | | | | | |
| Miyake et al. | 60 | 16.9 (95% CI NR) | NR | 33(55%) | 26(43.3%) | 2(3.4%) | 6.8 (95% CI NR) |
| **Abiraterone after enzalutamide (E-A)** | | | | | | | |
| Maughan et al. (2016)^56^ | 16 | 29.9 (95% CI 18.8-NR) | NR | 3(18.8%) | 2(12.5%) | 0(0%) | 13.0 (95% CI 10.3-21.2)  PSA-PFS: 12.3 (95% CI 8.9-20.5) |
| Yamada et al. | 14 | 9.1 (95% CI 5.6-12.5) | NR | 1(7%) | 1(7%) | 0(0%) | 3.4 (95% CI 0.8-6.0) |
| Terada et al. | 85 | 30.0 (95% CI 24.8-NR) | NR | 13(17.1%) | 10(13.1%) | 1(1.3%) | PSA-PFS: 9.9 (95% CI 7.8-11.9) |
| Matsubara et al. (2017)^57^ | 47 | 24.2 (95% CI 20.2-28.2) | NR | NR | 1(2.1%) | NR | 9.04 (95% CI 6.8-11.2) |
| Attard et al. | 125 | NR | 2.8 (95% CI NR) | 9(7.2%) | 3(2.4%) | 1(0.8%) | 5.6 (95% CI NR) |
| Khalaf et al. | 75 | 24.7 (95% CI 18.8-34.0) | 1.7 (95% CI 1.6-2.5) | 3(4%) | 2(2.7%) | 0(0%) | NR |
| Komura et al. | 38 | 14 (95% CI NR) | 3 (95% CI NR) | 5(13.9%) | 3(8.3%) | 0(0%) | rPFS: 7 (95% CI NR) |
| **Enzalutamide after docetaxel and abiraterone (D-A-E)** | | | | | | | |
| Thomsen et al. (2014)^34^ | 24 | 4.8 (95% CI 3.0–8.4) | NR | 11(46.9%) | 4(16.7%) | 0(0%) | NR |
| Badrising et al. (2013)^35^ | 61 | 7.9 (95% CI >7.2) | 4.4 (95% CI >4.0) | 28 (46%) | 13 (21%) | 2(3%) | 3.0 (95% CI 2.8–4.0) |
| Schmid et al. | 35 | 7.5 (95% CI 4.7-10.3) | NR | 13% | 10% | 0(0%) | 3.1 (95% CI 1.4-4.8) |
| Schrader et al. (2014)^46^ | 35 | 7.1 (95% CI 6.2–8.1) | 4.0 (95% CI 2.0-6.0) | 13(37.1%) | 10(28.6%) | 2(5.7%) | NR |
| Bianchini et al. (2014)^37^ | 39 | NR | 2.7 (95% CI 2.5-3.0) | 16(41%) | 5(13%) | 1(2.5%) | 2.8 (95% CI 2.0–3.7) |
| Brasso et al. | 137 | 8.3 (95% CI 6.8–9.8) | 6.7 (95% CI 5.5-7.9) | 45 (38%) | 22 (18%) | 3（2.5%） | 3.1 (95% CI 2.3–3.9) |
| Cheng et al. | 165 | 12.2 (95% CI 10.7- 16.5)  12-month OS: 51% (41%–62%) | NR | 40(24%) | 28(17%) | 4(2%) | PSA-PFS: 2.8 (95% CI 2.5–3.2) |
| Azad et al. | 68 | 10.6 (95% CI NR) | NR | 24(35.3%) | 15(22%) | 4(5.9%) | NR |
| **Abiraterone after enzalutamide and docetaxel (D-E-A)** | | | | | | | |
| Loriot et al. | 38 | 7.2 (95% CI 5.0–NR) | NR | 7(18%) | 3(8%) | 0(0%) | 2.7 (95% CI 2.3– 4.1) |
| Noonan et al. | 30 | 11.6 (95% CI 6.5–16.6) | 3.9 （95% CI 2.7-5.0） | 3(11%) | 1(3%) | 0(0%) | 3.9 (95% CI 2.7–5.1) |

Abbreviations: 95% CI, 95% confidence interval; OS, overall survival; PSA, prostate-specific antigen; PSA-PFS, PSA-progression-free surviva; rPFS, radiographic progression-free survival; NR, not reported.
