# Supplementary figures and images for: Abiraterone, Orteronel, Enzalutamide and Docetaxel: Sequential or Combined Therapy?
Source: Front Pharmacol. 2022 Feb 17;13:843110. doi: 10.3389/fphar.2022.843110 (PMC8891580; doi:10.3389/fphar.2022.843110)

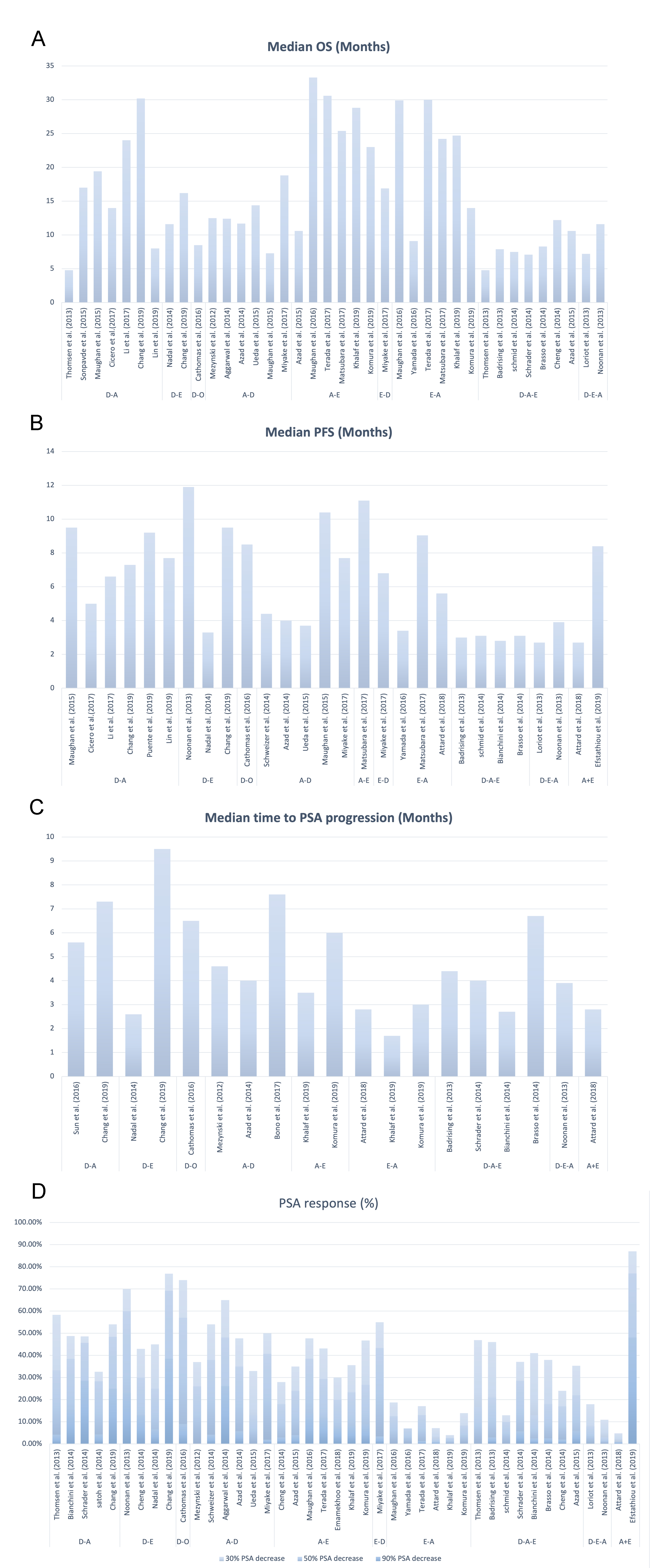

Supplement: Supplementary file 2 [file Image1.jpeg]
