# Supplementary material for: Abiraterone, Orteronel, Enzalutamide and Docetaxel: Sequential or Combined Therapy?
Source: Front Pharmacol. 2022 Feb 17;13:843110. doi: 10.3389/fphar.2022.843110 (PMC8891580; doi:10.3389/fphar.2022.843110)
Supplement: Supplementary file 3 [file Table2.docx]

Supplemental table 2. Clinical Outcomes of Combined Treatment in Metastatic Castration-Resistant Prostate Cancer

|  | **Sample Size** | **Median OS (Months)** | **Median time to PSA progression (Months)** | **PSA response (%)** | | | **Median PFS (Months)** |
| --- | --- | --- | --- | --- | --- | --- | --- |
|  |  |  |  | **30% PSA decrease** | **50% PSA decrease** | **90% PSA decrease** |  |
| **Abiraterone with enzalutamide (A+E)** | | | | | | |  |
| Attard et al. | 126 | NR | 2.8 (95% CI NR) | 6(4.8%) | 1(0.8%) | 1(0.8%) | 5.7 (95% CI NR) |
| Efstathiou et al. | 60 | NR | NR | 52(87%) | 46(77%) | 29(48%) | 8.4 (95% CI 4.9-11.2) |

Abbreviations: 95% CI, 95% confidence interval; OS, overall survival; PSA, prostate-specific antigen; NR, not reported.
